# Endogenous opioids facilitate intrinsically-rewarded birdsong

**DOI:** 10.1038/s41598-020-67684-1

**Published:** 2020-07-06

**Authors:** Sharon A. Stevenson, Alice Piepenburg, Jeremy A. Spool, Caroline S. Angyal, Allison H. Hahn, Changjiu Zhao, Lauren V. Riters

**Affiliations:** 1 Department of Integrative Biology, University of Wisconsin Madison, 428 Birge Hall, 430 Lincoln Drive, Madison, WI 53706 USA; 2 Department of Psychological and Brain Sciences, University of Massachusetts Amherst, Amherst, MA 01003 USA; 3 Department of Psychology, St. Norbert College, De Pere, WI 54115 USA

**Keywords:** Motivation, Animal behaviour

## Abstract

Many songbirds sing in non-reproductive contexts while in flocks. Singing in such gregarious contexts is critical for maintaining and learning songs; however, song is not directed towards other individuals and has no obvious, immediate social consequences. Studies using conditioned place preference (CPP) tests of reward indicate that song production in gregarious contexts correlates positively with a bird’s intrinsic reward state and with opioid markers in the medial preoptic nucleus (mPOA). However, the causal involvement of opioids in gregarious song is unknown. Here we report that the selective mu opioid receptor (MOR) agonist fentanyl dose-dependently facilitates gregarious song and reduces stress/anxiety-related behavior in male and female European starlings. Furthermore, infusion of siRNA targeting MORs specifically in mPOA both suppresses gregarious song and disrupts the positive association between affective state and singing behavior, as revealed using CPP tests of song-associated reward. Results strongly implicate opioids in gregarious song and suggest that endogenous opioids in the mPOA may facilitate song by influencing an individual’s intrinsic reward state.

## Introduction

Primary functions of birdsong include mate attraction and territory defense^1,2^. In these reproductive contexts, songs are directed towards potential mates or rivals and can be reinforced immediately by copulation or the departure of a rival. Although less studied, many birds also engage in high rates of singing behavior outside these primary contexts. For example, singing behavior in large flocks outside the context of breeding is critical for vocal learning and maintenance^3–6^. Despite evidence that singing behavior outside primary contexts plays an important role in the natural life history of songbirds, the immediate consequences of song in such contexts are not clear. Specifically, individuals do not appear to direct vocal signals towards particular individuals, and songs appear to be ignored by potential recipients^7–10^.

In the absence of any obvious immediate social consequences, it has been proposed that vocal behavior in non-sexual contexts is intrinsically facilitated and reinforced by an individual’s reward state^6,11–15^. Indeed, it was proposed by Darwin that birds sing “for their own amusement after the season for courtship is over” ^16^, and recent studies in songbirds support this hypothesis. Songbirds (including our experimental species, European starlings, *Sturnus vulgaris*) produce high rates of learned communication in non-sexual, gregarious contexts. Specifically, outside the context of mating, male and female starlings form large flocks in which they sing at high rates, share song types, display little aggression, and share perches and food sources^17,18^. Male starlings modify the structure of song in this “gregarious” context such that it contains features that are less attractive to females and non-threatening to other males, which may play a role in promoting social tolerance^19–21^. However, birds do not appear to direct song towards any particular individual and there is no evidence that this type of song is reinforced extrinsically by the behavioral responses of conspecifics. That is, it does not immediately attract mates or repel intruders^6,17,18^. In past studies, using a conditioned place preference (CPP) test to measure song-associated reward we found that gregarious song is tightly coupled to an intrinsic reward state^12,22,23^. Specifically, starlings show a preference for a chamber that they were exposed to immediately after singing, suggesting that the reinforcing internal state associated with singing becomes positively coupled with contextual cues experienced around the time of singing.

Opioid neuropeptides that specifically bind to mu opioid receptors (MORs) induce reward / a positive affective state^24,25^. Multiple studies implicate opioids in social reward (e.g., ^26–31^), and past research in songbirds strongly implicates opioids, and specifically MOR in the medial preoptic nucleus (mPOA), in gregarious song^6,9,23,32–35^. For example, the non-selective opioid receptor antagonist naloxone reduced “undirected” song (i.e., a form of non-sexual, song common in flocks) in male zebra finches^36^. In starlings, an indirect measure of opioid release and opioid markers in the mPOA were linearly, positively associated with the amount of gregarious song produced^23,33,37,38^. Furthermore, positive correlations were found between gregarious song, a bird’s affective state (measured using CPP), and mRNA measurements in mPOA for MOR as well as the precursor for a MOR ligand, pre-proenkephalin^23^. These correlational data strongly suggest that gregarious singing behavior, reward state, and opioid markers are related; however, the causal nature of these relationships has not been tested.

In the present study, we report that peripheral pharmacological treatment with the selective MOR agonist fentanyl dose-dependently stimulated gregarious song in male and female starlings. Additionally, downregulation of MOR in mPOA via siRNA infusions both suppressed gregarious song and disrupted the development of a song-associated CPP. These results demonstrate a central role for MOR in gregarious singing behavior and suggest that MORs in the mPOA may facilitate song by influencing the affective state associated with singing behavior.

## Results

### MOR agonism increased gregarious singing and decreased stress-associated behavior

We treated female (n = 7) and male (n = 7) starlings housed in outdoor, same-sex flocks peripherally with saline or the MOR agonist fentanyl to test the hypothesis that stimulation of MORs increases gregarious singing. Because males and females were tested during different years in different flocks (see methods), we did not directly statistically compare the males to the females (as differences in the testing environment or weather would be conflated with potential sex differences).

**Figure 1:**
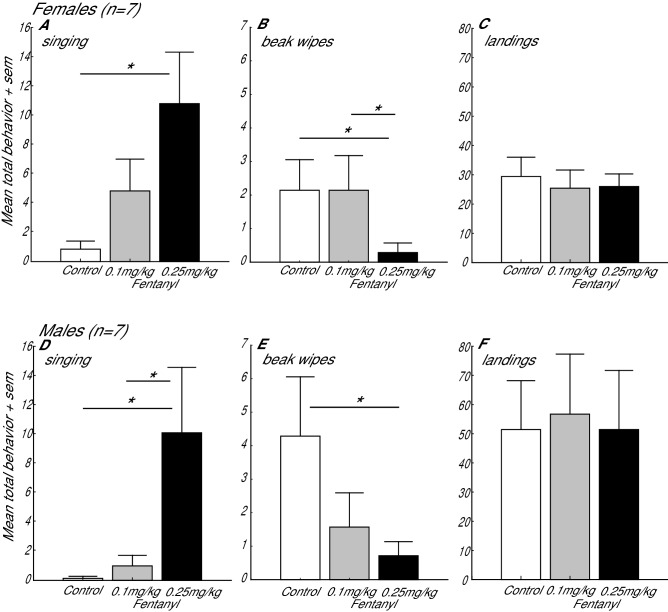
The selective MOR agonist fentanyl increased singing behavior and decreased beak wiping. Mean total singing behavior, beak wipes (a reflection of anxiety / stress), and landings in females (**A**–**C**) and males (**D**–**F**) in birds receiving subcutaneous injections of control (open bars), 0.1 mg/kg (gray bars), and 0.25 mg/kg (black bars) fentanyl. * indicates *p* < 0.05.

Separate repeated measures ANOVAs on the female and male data sets revealed that fentanyl significantly increased singing behavior relative to control treatment (females: F (2,12) = 5.36, *p*  = 0.022; males: F (2,12) = 4.68, *p* = 0.032). Posthoc Fisher’s LSD analyses revealed that females sang significantly more after the high dose fentanyl treatment compared to control treatment (*p* = 0.007), and males sang more after the high dose of fentanyl treatment compared to the control (*p* = 0.017) and low dose fentanyl treatments (*p* = 0.027) (Fig. 1A,D).

To provide insight into the possibility that opioid release may also suppress negative internal states that inhibit gregarious singing, we examined beak wiping, which in starlings is associated with stress^39,40^. Fentanyl treatment significantly reduced beak wiping behavior in females with a similar trend for males (repeated measures ANOVA; females: F (2,12) = 4.69, *p* = 0.0312; males: F (2,12) = 2.83, *p* = 0.099; Fig. 1B, E). Posthoc Fisher’s LSD analyses revealed that females performed less beak wiping after the high dose fentanyl treatment compared to either control treatment (*p* = 0.021) or low dose fentanyl treatment (*p* = 0.021), and males performed significantly less beak wiping after high dose fentanyl treatment compared to control treatment (*p* = 0.042). To test the possibility that fentanyl was affecting motor behavior, we examined the number of times birds landed on perches in the aviary, and found no significant effects of treatment (repeated measures ANOVAs; females: F (2,12) = 0.53, *p* = 0.601; males: F (2,12) = 0.04, *p* = 0.962; Fig. 1C,F).

### Effects of siRNA infusions on MOR mRNA and protein measurements

We next conducted an experiment to begin to causally test the role of MOR in mPOA in gregarious song. As part of this study we also ran a CPP test of song-associated reward. We reasoned that if MOR activity underlies reward associated with gregarious song, then siRNA downregulation targeting MOR in mPOA should reduce singing behavior and also break the link between singing and CPP observed in previous studies (reviewed in the introduction).

**Figure 2:**
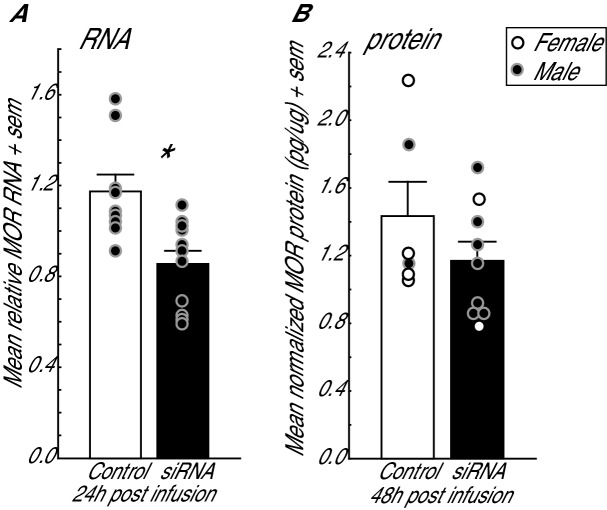
Mean measurements of MOR mRNA (**A**) and protein (**B**) in punches taken from mPOA in birds infused with with negative control sequences (open bars) or siRNA sequences targeting MOR (filled bars) in mPOA. mRNA was measured 24 h after infusion in a group of untested males to validate the method. Protein was measured 48 h after infusion in control and experimental males and females to determine the extent of downregulation in birds immediately after completion of the CPP test. Dots are provided to illustrate male and female data. Dots represent individual birds; open dots = females; filled dots = males. * indicates *p* < 0.05.

The effectiveness of siRNA manipulations to downregulate MOR in mPOA was tested at 24 and 48 h post-infusion. First, in a separate set of birds that was not behaviorally tested, we validated that the siRNA treatment downregulates MOR mRNA in the mPOA 24 h after siRNA infusion, when birds undergo the conditioning phase of the CPP paradigm (described below). This validation included only males because the goal was simply to determine that the siRNA would be effective at downregulating MOR at this time (see methods). We found that at 24 h post-infusion mean relative MOR mRNA in mPOA was significantly lower in siRNA treated birds than in controls, confirming downregulation at this time (Fig. 2A; one-tailed t_18_ = 3.42, *p* = 0.002; two-tailed *p* = 0.004; we report a one-tailed t-test because siRNA is predicted to impact MOR mRNA in only one direction [i.e., downregulate] but to be complete, we also report the two-tailed).

We also measured MOR protein 48 h after infusions in the birds that were tested behaviorally. We found that mean normalized MOR protein concentration in mPOA was on average lower in siRNA treated birds than in controls, but this was not statistically significant (Fig. 2B; one-tailed t_13_ = 1.24, p = 0.118; two-tailed *p* = 0.2369). We note that MOR protein did not differ between the control males (n = 2) and females (n = 4) (one-tailed t_4_ = 0.22, *p* = 0.836). This suggests that there were no sex differences in baseline MOR.

### Sex and behavior

On habituation day, prior to infusion with siRNA or control solution, t-tests revealed no significant differences between males (n = 10) and females (n = 6) in singing behavior (t_14_ = 0.78, *p* = 0.448), beak wiping (t_14_ = 0.28, *p* = 0.784) or feeding + drinking (t_14_ = 0.22, *p* = 0.829). We also note that our post-treatment groups included only 2 females in the siRNA group and 2 males in the negative control group. Thus, although we show individual male and female data points in figures, we ran final analyses with the sexes combined.

### MOR downregulation, song, and song-associated CPP

**Figure 3:**
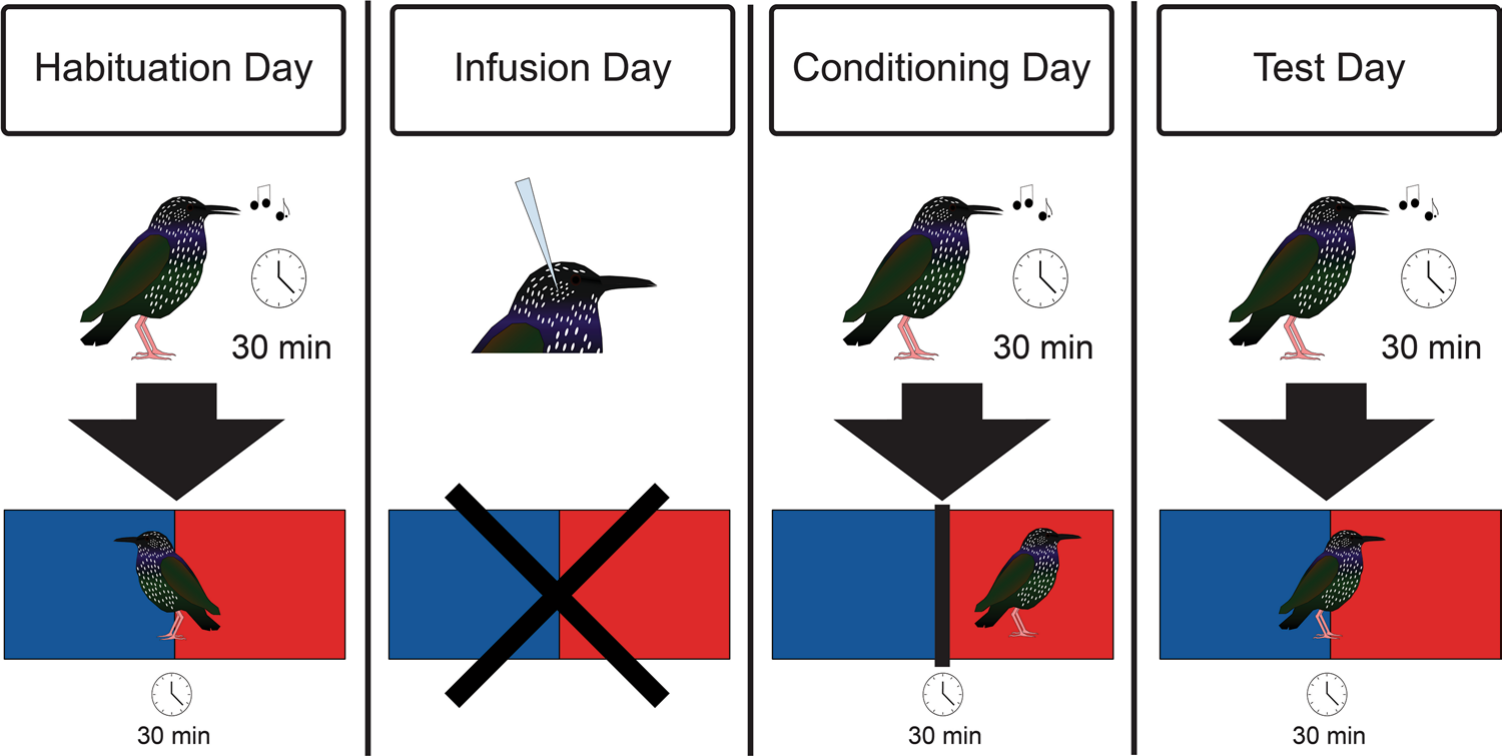
Overview of the CPP test for song-associated reward. *Habituation Day*, birds sang for 30 min and then explored the CPP chamber consisting of a red and blue compartment for 30 min. *Infusion Day*, birds received an infusion targeting mPOA. *Conditioning Day* (24 h post infusion), birds were confined to one of the distinct compartments immediately after singing. *Test Day* (48 h post infusion), birds sang and then the time spent in the 2 compartments was recorded for 30 min.

The CPP test took place over 4 days (Fig. 3). Briefly, on Day 1, *Habituation Day*, birds sang for 30 min and were then habituated to the CPP apparatus by freely exploring its two distinct chambers for 30 min. On Day 2, *Infusion Day*, birds received a control or siRNA infusion targeting mPOA. On Day 3, *Conditioning Day* (24 h post infusion), birds were confined to one of the two distinct compartments immediately after singing. On Day 4, *Test Day* (48 h post infusion), birds sang and then were given 30 min to freely explore the apparatus. Several previous studies demonstrate that gregarious singing behavior on *Conditioning Day* is positively associated with birds choosing to spend more time in their *Conditioning Day* compartment on *Test Day*, reflecting an association between that affective state associated with gregarious singing and the compartment^12,22,23^.

**Figure 4:**
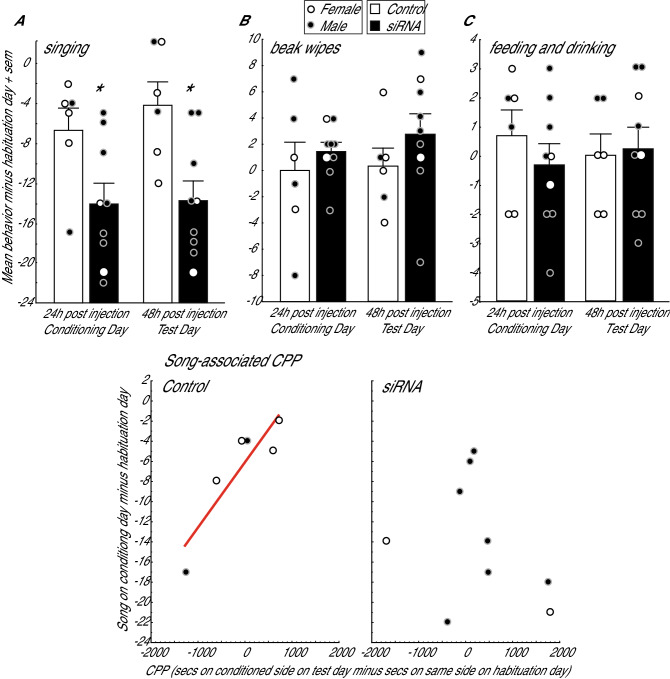
siRNA targeting MOR in mPOA reduces singing behavior 24 and 48 h post-infusion. Mean total singing behavior (**A**), beak wipes (**B**), and feeding plus drinking (**C**) in starlings infused with negative control sequences (open bars) or siRNA sequences targeting MOR (filled bars) in mPOA. * indicates *p* < 0.05. Dots are provided to illustrate data for females (open dots) and males (filled dots). Song measures are negative because birds always sing less after surgery, but the siRNA treated birds sang even less than controls. Lower figures: siRNA targeting MOR in mPOA disrupts the association between song and reward. Song correlated positively with reward state (measured using CPP, detailed in text) in birds infused with negative control sequences but not in birds infused with siRNA. Open dots = females, filled dots = males. The presence of a regression line indicates *p* < 0.05.

siRNA treated birds sang less than controls on both conditioning day (24 h post infusion) and test day (48 h post infusion), but beak wiping and feeding + drinking were unaffected (Fig. 4A–C). A General Linear Model (GLM) analysis with treatment (i.e., siRNA or control treatment) entered as an independent variable and song, beak wipe and feeding + drinking entered as non-independent dependent variables revealed a significant treatment x behavior interaction at both 24 and 48 h (24 h: F_2,26_ = 3.45, *p* = 0.047; 48 h: F_2,26_ = 8.96, *p* = 0.001). Posthoc Fisher’s LSD analysis revealed that siRNA treated birds sang significantly less than controls (24 h: p = 0.0009; 48 h: *p* = 0.003; Fig. 4A). siRNA treated birds did not differ from controls in measures of beak wiping (24 h: p = 0.41; 48 h: *p* = 0.36) or feeding + drinking (24 h: *p* = 0.57; 48 h: *p* = 0.93; Fig. 4B,C). A significant main effect for treatment was also found at 24 h (F_1,13_ = 5.43, *p* = 0.037) but not 48 h (F_1,13_ = 2.38, *p* = 0.147). We note that behaviors did not differ between the control and siRNA infused birds prior to treatment as measured during the habituation period (treatment: F_1,13_ = 0.52, *p* = 0.485; treatment x behavior interaction: F_2,26_ = 2.08, *p* = 0.145). This confirms that behaviors did not differ in birds in the two groups prior to treatment.

siRNA targeting MOR in mPOA also broke the positive association between singing and CPP. We used the number of songs produced on Conditioning Day minus the number of songs produced on Habituation Day as the measure of singing behavior to take into account individual baseline differences in behavior. Birds in the control condition developed a song-associated CPP as indicated by a significant positive correlation between the measure of singing behavior and the amount of time birds spent in the previously-song paired chamber of the CPP apparatus on Test Day (r = 0.89, *p* = 0.019; Fig. 4). In contrast, there was no significant correlation between song behavior and time in the previously-song paired chamber for birds infused into mPOA with siRNA targeting MOR (r = −0.31, *p* = 0.412; Fig. 4). A homogeneity of slopes analysis revealed that the slopes of the correlations for the control and siRNA treated groups differed significantly (*p* = 0.044).

To provide insight into the degree to which the CPP was selectively associated with singing behavior, we ran correlations between the time spent on Test Day in the previously song-paired chamber and measures of both beak wiping and feeding + drinking (on conditioning day minus the measures on Habituation Day, as for singing behavior). No significant relationships were identified between the place preference measure and either behavioral measure for either control or siRNA treated birds (beak wiping: control r = −0.14, p = 0.792; siRNA, r = −0.35, *p* = 0.350; feeding + drinking: control r = −0.46, *p* = 0.364; siRNA, r = 0.46, *p* = 0.208). Furthermore, if the affective state associated with singing immediately prior to being placed into a CPP chamber leads to the preference for that chamber, then singing behavior produced at other times (e.g., on Test Day) would not be expected to correlate with time spent on Test Day in the previously-song paired chamber. We ran this analysis and found no significant correlations between the place preference measure and song on Test Day (control r = 0.46, *p* = 0.364; siRNA, r = −0.19, *p* = 0.455).

## Discussion

This study is the first to demonstrate a causal role for MOR in gregarious song in both male and female songbirds. Results also suggest a role for MOR in mPOA in gregarious song and the positive affective state associated with singing behavior. More broadly, this study is consistent with a growing body of research that expands what is known about the role of mPOA to include an important role in non-sexual social behaviors, a role that may generalize to other species and other intrinsically-rewarded social behaviors as detailed below.

### MOR agonism reduces stress/anxiety and stimulates gregarious singing behavior

We report that peripheral injections of the selective MOR agonist fentanyl cause dose-dependent increases in gregarious singing behavior in male and female starlings, while at the same time decreasing beak wiping, which is considered a sign of stress or anxiety^39,40^. The fentanyl did not affect landings, indicating that fentanyl effects on behavior were not caused by gross deficits in motor activity.

Past studies demonstrate that opioids act at MOR to induce both a positive affective state and to reduce signs of anxiety or fear^41–46^. Gregarious singing behavior is facilitated by the presence of flock mates when birds are free from stress and fear (e.g., in the absence of predators) ^18,47–49^. Thus our findings suggest that the presence of flock mates/safety of a flock may naturally lead to opioid release and stimulation of MOR to induce a positive/low anxiety state that is conducive to gregarious singing behavior. This idea is further supported by the observation that in both the female and male studies, control birds sang very little. Given that birds were only selected for inclusion in the study if they sang at high rates consistently during pre-test observations, this suggests that the injection procedure, which involves chasing, catching, and injecting the bird, induced a state of stress/anxiety in the birds that reduced singing behavior. The finding that fentanyl rescued singing behavior is consistent with the idea that natural MOR stimulation may facilitate song by reducing a state of stress or anxiety. It may also be that opioids are released naturally by the act of singing itself to maintain ongoing singing behavior.

It is noteworthy that in past studies the highest dose of fentanyl (0.25 mg/kg), which we report here to stimulate gregarious song, inhibited sexually-motivated song in male starlings^50^. These opposing findings are consistent with studies of intrinsically-rewarded social behavior and extrinsically-rewarded, mate-directed behavior in mammals. Specifically, stimulation of MOR in rats facilitates gregarious social play behavior (behavior that, similar to gregarious song, has been shown to be intrinsically-rewarded using CPP tests^27,31,51–53^). In contrast, MOR agonists in male rats generally inhibit mate-directed behaviors that can be extrinsically-rewarded by copulation^54–60^, as in our past study on sexually-motivated song in male starlings^50^. The similarities identified in the roles played by opioids in gregarious and sexual behaviors in songbirds and rats, suggest that mechanisms of opioid action on socio-sexual behaviors may be conserved across species. Furthermore, the finding that MOR agonism affected behaviors similarly in males and females suggests that similar mechanisms may underlie this type of singing behavior in both sexes.

### MOR downregulation disrupts singing behavior and associated reward

siRNA targeting MOR in mPOA suppressed gregarious song. Specifically, relative to birds infused with negative control sequences, birds infused with siRNA sang significantly less both 24 h and 48 h post infusion. Unlike the peripheral MOR manipulations, this treatment did not significantly impact beak wiping, indicating that MOR acts in other brain regions (such as the periaqueductal gray^21^) to reduce stress/anxiety needed to facilitate gregarious song. No effects were observed on feeding or drinking behaviors, suggesting that siRNA treatment did not induce non-specific changes in behavior. (Although MOR are involved in feeding behavior^61,62^, the mPOA has not been identified as a critical site of action for this behavior.)

Because infusion of opioids that stimulate MOR into the mPOA induces reward in rats^63^, we hypothesized that MOR in mPOA may underlie the reward state associated with gregarious song in songbirds. Consistent with this hypothesis, siRNA targeting MOR in mPOA disrupted the positive correlation observed in controls between singing behavior and a positive affective state, measured using CPP. Specifically, for control birds song rate correlated positively with the amount of time a bird spent in a chamber in which it had been placed previously after singing. Thus these birds demonstrated a song-associated CPP. In contrast, there was no positive relationship between song rate and CPP in siRNA treated birds. We interpret these results as consistent with a role for MOR in mPOA in the positive affective state associated with singing behavior; however, other interpretations are also possible. For example, the lack of correlation in MOR knockdown birds may be interpreted to reflect learning or memory deficits rather than a lack of reward. Although we found no publications implicating MOR in mPOA in learning and memory, such alternative interpretations must be considered.

We interpret our findings cautiously because in this study the sample size for control birds was limited; however, the positive relationship between song and CPP observed in the controls here replicates results of four prior studies from our lab (including one in male zebra finches) that used this method to assess song-associated reward^12,22,23^, suggesting the correlation in controls is not likely due to chance. Although sample sizes for the sexes were not large in this study, these initial findings do not demonstrate sex differences, suggesting that mechanisms rewarding gregarious song may be similar across males and females, but this must be tested in future studies.

### Additional considerations related to siRNA methodology

Our initial validation study demonstrates strong suppression of MOR mRNA in mPOA at 24 h. After using an identical infusion protocol for the behavioral study, we see the same pattern for MOR protein 48 h post-infusion; however, MOR protein was not significantly lower in siRNA treated birds compared to controls. This lack of a significant difference is not unexpected given individual differences in MOR are likely present in individuals prior to treatment and because MOR synthesis may begin to be restored at the time that brains were collected. Thus, given the effectiveness of the method at 24 h we assume that we did successfully knockdown MOR receptors in the mPOA at the time of conditioning in the birds used in the behavioral experiment.

A second caveat is that we do not know how far the siRNA infusions spread. It is likely that infusions spread and downregulated MOR beyond the boundaries of mPOA. Thus, it is possible that effects on behavior are caused by MOR downregulation outside mPOA. Although possible, past studies that show that lesions to mPOA nearly abolish spring song also show that lesions located even slightly outside the boundaries of mPOA have no effect^19,64,65^. Similarly, studies that demonstrate strong effects of intra-mPOA opioid and dopamine receptor manipulations on singing behavior also show no effects on behavior when cannulae miss mPOA, even by a small margin^38,66^. Finally, in songbird studies MORs appear to be denser in mPOA than in the immediately surrounding regions^67,68^. Altogether these past studies suggest that the area immediately surrounding mPOA is not a primary site involved in the regulation of song but do not preclude the possibility that MOR downregulation in sites outside of mPOA may have influenced behavior.

### Additional considerations related to the CPP methodology

Our attempt to assess rewarding properties associated with the act of producing song presents unique challenges that require modification of the CPP methodology somewhat from CPP tests used in studies of drug or food reward (reviewed^69,70^). Unlike food or drugs we cannot administer the “act of singing”. Birds either sing or they do not, which means that we cannot pair the act of singing with one chamber in a CPP apparatus and a lack of singing with another chamber an equal numbers of times and then compare time spent in each chamber to a neutral zone, as is common in studies of food or drug reward. This imbalanced design causes birds to be exposed to the song-paired side of the apparatus more than the non-song-paired side. Thus, it may be that on test day something about the more familiar side of the CPP apparatus (i.e., the previously song-paired side) is more appealing to birds singing higher rates of undirected song. Although we interpret our results cautiously, without a compelling reason to propose that high singers should be more attracted to familiarity than low singers, we suggest that a straightforward interpretation of the results is that gregarious singing behavior is coupled to a positive affective state. Detailed discussion of this and other interpretational issues can be found in^12^.

## Conclusions and broader implications

Neural systems that underlie important social behaviors are often conserved across species^71–73^. This suggests that studies of gregarious communication in songbirds may be uncovering a core, conserved circuit in which opioids act at MOR in mPOA to stimulate, maintain, and reward important social behaviors in contexts in which there is no obvious extrinsic reward. We recently tested this hypothesis by exploring a role for MOR in mPOA in social play in rats, which is a well-studied form of intrinsically-rewarded social behavior. We demonstrated that the immediate early gene egr-1 is higher in mPOA in rats that are permitted to play compared to those that are isolated, that egr-1 labeled cells in mPOA of playing rats co-localize with MOR protein label, and that shRNA downregulation of MOR in mPOA suppresses play behavior^74^. The results of this rat study indicate that studies of the mPOA in songbirds are uncovering a central nucleus that is part of a core, conserved circuit in which opioids act to facilitate important social behaviors in contexts for which there is no obvious extrinsic reward. By revealing core, conserved circuits underlying gregarious communication, studies in songbirds have the potential to provide novel ideas for treatments to restore positive social interactions in humans with mental health disorders.

## Materials and methods

All procedures followed protocols approved by the University of Wisconsin Madison Institutional Animal Care and Use Committee and were performed in accordance to guidelines and regulations approved by the *National Institutes of Health Guide for the Care and Use of Laboratory Animals*. Data generated and analyzed as part of this study are available on request.

### Peripheral pharmacological MOR manipulations

Fourteen female and seventeen male adult European starlings were used. Each bird was assigned a unique combination of colored leg bands for individual identification. Birds were trapped on a local farm in winter and housed in indoor aviaries under a photoperiod of 18 h light until being transferred to outdoor observation aviaries in August and September for the experiment. Each aviary (2.13 m × 2.4 m × 1.98 m) contained multiple branches for perching, nesting boxes, a bath, food (chick starter) and drinking water. Flocks of female starlings were observed in fall 2017 and flocks of male starlings were observed in fall 2018.

For the female study, birds were moved into 2 outdoor aviaries (7 females/aviary). For the male study, birds were moved into 4 outdoor aviaries (4 birds in three aviaries and 5 in one). Birds were habituated to the presence of an observer behind a blind on approximately 3 days each week in August and September. Behavioral observations began as soon as most birds in each aviary were singing and continued until 7 males and 7 females had been tested. Testing took place from October to December for both the male and female studies.

A round of testing started with the selection of a focal bird that had been observed singing at high rates (i.e., birds singing approximately 6–10 times in a 20 min observation period on at least 2 days). The focal bird was injected with vehicle solution (dH_2_O; 0.05 ml into the inguinal leg fold) and released back into the aviary to habituate on the day prior to experimental testing. The pharmacology study began two days after this injection. Birds were tested on 3 test days that were separated by at least one day. The focal bird was injected into the inguinal leg fold with either control solution (dH_2_O; 0.05 ml), 0.10 or 0.25 mg/kg fentanyl (Sigma; F-3886; 0.05 ml dissolved in dH_2_O) on each test day. Birds received a different treatment on each test day with the order counterbalanced across birds. Birds were immediately released back into the aviary after injections. Fifteen minutes after injection, an observer who was unaware of treatment conditions observed the focal bird from behind a blind for 15 min. Starling flock song is facilitated by the presence of other singing birds. Numerous birds were singing in the focal or neighboring aviaries during testing on most days; however, if no birds were singing, at the onset of testing the experimenter played recordings of male starling song for 5 min (or until birds began to sing). Playback did not correspond to any particular treatment condition (i.e., playback was equally likely to be used after birds were treated with saline or one of the two doses of fentanyl).

During each observation period, the observer noted multiple behaviors. We focus here on three: (1) *Singing*: # of total songs (fragments + introductory whistles + full songs), (2) *beak wiping*: a potential sign of stress in starlings^39,40^, (3) *# of times a bird flew to and landed on a new perch:* a non-specific measure of motor activity. Birds were rarely observed feeding or drinking and agonistic interactions occurred too infrequently to analyze. After completing analysis of one focal bird, a new bird was selected for observation during the next round of testing.

### Site-directed siRNA manipulations, gregarious song, and CPP

Quantitative real time PCR (qPCR) was first used to establish the effectiveness of our siRNA procedure to downregulate MOR mRNA in mPOA. We included only males in this preliminary study because the goal was simply to determine effectiveness of the siRNA to downregulate MOR. In addition, by using only males we were able to reduce any confounds that may be caused by potential sex differences in MOR mRNA. Twenty adult male starlings were anesthetized using isoflurane and placed into a stereotaxic apparatus (Kopf Instruments). A cannula lowered to the coordinates for mPOA (detailed below) was used to infuse birds with MOR siRNA to downregulate MOR (n = 11) or negative control sequences (n = 9) following the stereotaxic procedures described below. The MOR siRNA (Stealth siRNA, Invitrogen) contained 6 sequences (3 forward and three reverse) targeting the mu opioid receptor gene (OPRM1). The negative control infusions contained 6 sequences that match no known avian genes but matched the approximate G/C-content of the siRNA (Table 1).

The stereotaxic apparatus was set with the beak approximately 45° below the plane of the ear bars. The rostral/caudal zero point was established by placing the cannula in the apparatus and lowering the cannula to the middle of the ear bar in all planes. The initial horizontal point was set to − 0.7 mm from zero. Ear bars were placed in the most rostral (anterior) ear position possible. A small incision was made to expose the skull, and the cannula lowered to the skull at the center of the mid vein, which can be seen through the skull immediately upon exposure. Both the lateral and vertical zeros were established at this position. The lateral target was placed + 0.6 mm from skull zero, to the right and left of the mid vein. A hole was drilled and the vertical target was lowered − 6.5 mm from skull zero.

siRNA/invivofectamine 2.0 complexes (Life Technologies) for both experimental and control infusions were prepared as described by the manufacturer. Briefly, 50 μL siRNA (2.4 μg/μL) was combined with 50 μL complexation buffer before being added to 100 μL invivofectamine 2.0 reagent and mixed by vortexing. The siRNA/invivofectamine 2.0 complex was then incubated for 30 min at 50 °C. Finally, the resulting complex was diluted with 1 ml sterile saline to a total of 0.12 μg/μL complexed siRNA. The complexed siRNA and control samples were aliquoted into 10ul tubes and stored at −20 °C until used. The Aliquots were not refrozen or reused. An 8 mm cannula (PlasticsOne, C315I/SPC INTERNAL 39,877 33GA, Roanoke, VA) connected to a 30 cm piece of polyethylene tubing (PlasticsOne C232CT) was lowered to the target location and 0.5 μl MOR siRNA or matched volume control solution was infused bilaterally into 3 locations for each hemisphere of mPOA (separated by 0.30 μm along the anterior–posterior axis), beginning at −0.7 and ending at −0.1 from the ear bar zero. Infusions were made over 2 min and confirmed by following movement of an air bubble in the tubing to ensure flow. Each bird received 72 ng per hemisphere, for a total of 144 ng. The cannula remained in place for 3 min after each injection. After recovery, each bird was returned to its cage. Twenty-four hours later birds were rapidly decapitated. Brains were immediately removed and frozen in isopentane over dry ice.

Brains were sectioned using a cryostat at 250 microns. After the mPOA appeared ventral to the tractus septomesencephalicus, a Fine Science Tools Sample Corer (Item No. 18035–02; Foster City, CA, USA) was used to collect a single 2 mm diameter punch, centered on the midline, containing the mPOA. qPCR was used to measure MOR mRNA relative to two reference genes, peptidylprolyl isomerase A (PPIA) and phosphoglycerate kinase 1 (PGK1), to normalize variation in endogenous mRNA following the protocol and using the primers detailed in prior studies^75^. As detailed in the results (Fig. 2A), the siRNA significantly downregulated MOR in mPOA at 24 h (i.e., on Conditioning Day, Fig. 3) so we next examined the effects of this manipulation on behavior.

### siRNA infusion and CPP testing

Twenty-seven (14 female and 13 male) adult European starlings were trapped, assigned distinct combinations of colored leg bands for identification, and housed in cages as in the peripheral pharmacology experiment. Birds were placed on a photoperiod of 18 h light for at least 6 weeks, which induces a state of photorefractoriness that is characteristic of early fall, when starlings begin to sing in flocks^18,76^. Starlings were then transferred to indoor observation aviaries for the experiment. Each aviary (approximately 2 m × 2 m × 2.5 m) had a one-way glass for observations and contained multiple branches for perching, a bath, food and drinking water. In this study, males and females were housed in mixed-sex flocks (which matches what occurs naturally in fall and winter) in one of 5 aviaries. Each flock started with 5–6 birds (with either 2:3 or 1:2 female:male sex ratio) and flock size was reduced as birds were tested and removed from the aviaries (detailed below). Behavioral observations began as soon as most birds in each aviary were singing and continued until 9 males and 6 females had been tested. Fewer females than males were observed to sing, so fewer females were selected for testing.

Song-associated conditioned place preference (CPP) testing (Fig. 3) began with the selection of a single focal bird from each aviary that had been observed singing for several days. On the first day of the experiment (Habituation Day; Fig. 3), focal birds were observed in home aviaries for 30 min by a single observer stationed behind a one-way glass. The observer noted *singing behavior* and *beak wiping* as in Experiment 1. Birds rarely displayed agonistic behaviors (similar to Experiment 1); however, in this study birds were frequently observed drinking and feeding, thus the sum of *feeding and drinking* bouts (with a bout separated by ~ 2 s) was recorded as a non-specific measure of motor activity. The focal bird was then removed from the aviary and placed for 30 min into a CPP apparatus located in a separate room with a one-way glass. The CPP apparatus consisted of a standard bird cage (118 cm × 59 cm × 59 cm) divided into 2 chambers. One chamber was decorated with blue construction paper and the other chamber was decorated with red construction paper. There was no divider between the two chambers at this point. The amount of time the bird spent in each chamber was recorded by a single observer through a one-way observation glass to provide an index of an individual’s baseline (pre-conditioning) chamber preference. One day following habituation, each focal bird was anesthetized using isoflurane and placed into a stereotaxic apparatus. Birds were infused with either 0.5 µl MOR siRNA to downregulate MOR (n = 9; 2 females and 7 males) or control sequences (n = 6; 4 females and 2 males) following the protocol detailed above in the qPCR study (Infusion Day, Fig. 3).

Conditioning Day took place approx. 24 h after siRNA or control infusions. The behaviors of focal birds were first observed in home aviaries for 30 min by a single observer stationed behind a one-way glass. The observer noted singing behavior, beak wiping, and bouts of feeding and drinking as described above for the habituation period. Immediately after behavioral observations, each bird was rapidly captured and placed singly into a CPP apparatus and restricted by a divider to one of the 2 chambers for 30 min (Conditioning Day, Fig. 3) and then returned to its flock. The color of the chamber was counterbalanced across treatment conditions. Eight birds were placed into the red chamber and 7 were placed into the blue chamber balanced across treatment conditions.

Song-associated place preferences were tested 24 h after Conditioning (i.e., on Test Day, approx. 48 h after siRNA or control infusions; Fig. 3). Singing behavior, beak wiping, and bouts of feeding and drinking were recorded again for each focal bird for 30 min. The divider separating the chambers of the CPP apparatus was removed and each focal bird was placed singly into the center of the apparatus. The time each bird spent in each chamber was recorded for 30 min by a single observer through a one-way mirror. The rationale is that if singing behavior is associated with a positive affective state, then the bird will develop a positive association with the chamber in which it was placed immediately after singing and spend more time on that side of the apparatus on Test Day (i.e., it will develop a song-associated place preference). These methods are adjusted from some traditionally used methods^69,70,77–79^because starlings will not sing in fall condition in a conditioning chamber ^23^; however, they match methods used to study copulation-induced reward in rats^77,79^and have worked effectively in multiple studies of song-associated reward in our past work^12,22,23^. Immediately after testing (on Test Day), each bird was rapidly decapitated. Brains were removed, frozen in Isopentane, and stored at − 80C. After completing analysis of one focal bird a new bird from the same aviary was selected for observation during a next round of testing.

### Sandwich ELISA immunoassay to confirm MOR downregulation in siRNA treated birds

Brains were sectioned on a cryostat, and 250 micron thick micropunches were collected using a 2 mm diameter punch from mPOA as described above in the qPCR experiment. Tissue concentration of MOR protein in mPOA was measured to confirm downregulation in siRNA treated birds relative to controls using a commercially available enzyme-linked immunoassay (ELISA) kit (LS-F33204, LifeSpan BioSciences Inc, Seattle, WA, USA) according to manufacturer’s instructions. The intra- and inter-assay coefficiencies of variation were 8% and 10%, respectively. Total protein concentration was determined using BCA Protein Assay (Pierce Biotechnology, Rockford, IL, USA) as previously described^80^. The final concentration of MOR in each sample was calculated by normalizing MOR concentration against concentration of total protein and expressed as picogram per microgram protein.

**Table 1:** siRNA sequences used to downregulate MOR and negative control sequences.

	MOR siRNA sequences
1	UACAAAGGAUGGUACCGAAUGGCCA
2	UGGCCAUUCGGUACCAUCCUUUGUA
3	CAUGGCUCAAUUGACUGCACACUUA
4	UAAGUGUGCAGUCAAUUGAGCCAUG
5	AUAACAUGCGAACGCUCUUCAGGCG
6	CGCCUGAAGAGCGUUCGCAUGUUAU

	Negative control sequences
1	UGGUACUGGAUCCUACCUUUCCGUA
2	UACGGAAAGGUAGGAUCCAGUACCA
3	AAUGAGCGGAGAAGAAAGUACCUUG
4	CAAGGUACUUUCUUCUCCGCUCAUU
5	UUGAGCCGUACAAAUAUCACGGGCC
6	GGCCCGUGAUAUUUGUACGGCUCAA
